# Horizontal and Vertical Comparison of Microbial Community Structures in a Low Permeability Reservoir at the Local Scale

**DOI:** 10.3390/microorganisms11122862

**Published:** 2023-11-26

**Authors:** Zena Zhi, Ziwei Bian, Yuan Chen, Xiangchun Zhang, Yifei Wu, Hanning Wu

**Affiliations:** 1 State Key Laboratory of Continental Dynamics, Department of Geology, Northwest University, Xi’an 710069, China; zhizena@stumail.nwu.edu.cn (Z.Z.); 202110382@stumail.nwu.edu.cn (Z.B.); 2 College of Food Science and Technology, Northwest University, Xi’an 710069, China; y_chenyuan@163.com; 3 College of Biology and Agriculture, Zunyi Normal University, Zunyi 563006, China; l_spring_2004@126.com

**Keywords:** microbial-enhanced oil recovery, microbial community, low-permeability reservoir, vertical, horizontal

## Abstract

Petroleum microorganisms play a crucial role in the application of microbial-enhanced oil recovery, and the community structures of petroleum microorganisms have been widely studied. Due to variations in reservoir geological conditions, reservoir microbial communities exhibit unique characteristics. However, previous studies have primarily focused on microbial community changes within a single well, a single block, and before and after water flooding, and thus, cross-horizon and cross-regional comparative studies of in situ microbial communities are lacking. In this study, the 16S rRNA full-length sequencing method was adopted to study bacterial communities in crude oil samples taken from two wells at the same depths (depths of 2425 m and 2412 m) but approximately 20 km apart in the Hujianshan oilfield, located in the Ordos Basin. At the same time, the results were combined with another layer of research data from another article (from a depth of 2140 m). The aim was to compare the differences in the microbial community structures between the oil wells on a horizontal scale and a vertical scale. The results revealed that there were minimal differences in the microbial community structures that were influenced by the horizontal distances within a small range (<20 km), while differences were observed at a larger spatial scale. However, the dominant bacteria (*Proteobacteria* and *Bacteroidetes*) in the different oilfields were similar. Vertical depth variations (>300 m) had significant impacts on the communities, and this was mainly controlled by temperature. The greater the depth, the higher formation temperature, leading to an increase in thermophilic and anaerobic bacteria within a community.

## 1. Introduction

Oil reservoirs are typically extreme living environments characterized by high temperatures, high pressures, high salinity levels, and hypoxia [1,2]. Petroleum is very important to modern industry, and oil recovery can be divided into three stages: primary oil recovery, secondary oil recovery (water flooding), and tertiary oil recovery. Primary and secondary oil recovery use conventional oil recovery technologies [3]. Primary oil recovery utilizes the natural energy stored in a formation for extraction, but only 5–10% of the crude oil can be recovered. Once the oil is extracted using natural energy, the pressure within the pore spaces of a formation of rocks is relieved, resulting in a depletion of the reservoir’s energy. To replenish the formation energy to obtain higher crude oil recovery rates [4], most oilfields employ water injection. This technique can enhance production by 20–40% [5]. However, even after using conventional recovery methods (such as water flooding), there remains a significant amount of remaining crude oil in a reservoir, and this is estimated to be 50–70% [5,6]. Therefore, the extraction of residual oil plays a vital role in improving oil recovery.

Tertiary oil recovery, also known as enhanced oil recovery (EOR), can be categorized into thermal recovery and non-thermal recovery [6]. Thermal recovery is primarily applied in heavy oil recovery, whereas non-thermal recovery methods include chemical flooding, gas flooding, microbial-enhanced oil recovery (MEOR), and foam flooding. Chemical flooding and gas flooding are the main approaches used in tertiary oil recovery. Polymer flooding and surfactant polymer flooding (SP flooding) are considered the most well-established chemical flooding techniques [7]. Polymer flooding improves sweep efficiency, while surfactant flooding reduces residual oil. However, the incompatibility between fluids and formations can result in formation damage, reducing formation permeability and worsening reservoir connectivity [8]. For low and ultra-low permeability reservoirs, gas flooding is a more suitable exploration method [6,9]. CO_2_ gas dissolves in crude oil, forming a miscible phase, which enhances the mobility of the crude oil and increases its volume. CO_2_ gas flooding has been widely used in the United States because it has considerable CO_2_ reserves [10]. Although there are sufficient CO_2_ gas sources in China, CO_2_ needs to be treated before being injected into a well [6], which requires high-quality equipment and has high installation costs, and so there are fewer processed CO_2_ gas sources that can be used in such formations. The implementation process is costly, and the production and transportation of chemicals constitutes a significant expense. Furthermore, the uncontrollable and unpredictable CO_2_ leakage in a formation poses challenges [4]. All of these approaches pose potential risks to the environment. Microbial-enhanced oil recovery is a technology that leverages microorganisms and their metabolites to enhance oil recovery in reservoirs and crude oil [11]. Compared to traditional EOR technologies, MEOR is regarded as a low-cost and environmentally friendly technology [12]. Additionally, numerous studies have demonstrated its superior economic efficiency [13]. Microbial metabolites (e.g., biosurfactants, biopolymers, gas, acids, and solvents) can enhance crude oil fluidity and improve water spread efficiency by changing a reservoir’s wettability, reducing crude oil viscosity, degrading heavy components, and reducing interfacial tension (IFT). These modifications ultimately contribute to enhanced recovery [14,15,16].

A significant number of microorganisms in the reservoir that are capable of reproducing under extreme conditions are referred to as in situ microorganisms, and the activation of these microorganisms to facilitate their interactions with a reservoir and crude oil represent a crucial aspect of MEOR applications. The environments of oil wells vary extensively due to differences in origins, formation times, and burial depths. Different reservoirs exhibit distinct physical and chemical properties, such as depths, reservoir temperatures, pressure, pH levels, formation water salinity levels, etc., leading to variations in the compositions, growth habits, and necessary conditions of petroleum microbial communities [17]. Previous studies have indicated that temperature is considered to be the factor that most affects a microbial community [18,19]. Therefore, MEOR applications must be tailored and adjusted based on specific well characteristics. Gaining detailed knowledge of the structural characteristics of microbial communities in reservoirs is of the utmost importance for the success of MEOR. To achieve targeted and precise MEOR applications, it is crucial to compare the microbial community structures of different wells in both horizontal and vertical directions at local geographic scales.

Previous scholars have provided some understanding of petroleum microbial communities. According to Qi et al. [20], Ma et al. [21], and Zhang et al. [22], petroleum microorganisms mainly include hydrocarbon-oxidizing bacteria, saprophytic bacteria, anaerobic bacteria, etc. To achieve the goal of extraction, various microorganisms modify the properties of crude oil and reservoirs through processes such as emulsification, degradation, plugging, and others. The predominant bacteria found in reservoirs also exhibit certain commonalities. Zhang et al. [23], Tian et al. [24], and Tian et al. [25] demonstrated that the bacteria of the phyla *Proteobacteria* and *Bacteroidetes* were dominant in numerous oil reservoirs. Previous research has largely focused on a single well, a single block, or the differences in microbial communities before and after water flooding. However, there have been limited studies that have compared the changes in vertical and horizontal spatial microbial community structures, and this is mainly due to sampling restrictions and other factors.

Therefore, in this study, oil samples from two wells located at the same depths but approximately 20 km apart in the Chang 8 layer were collected as the research objects. Combined with the existing results of our team from the Chang 4 and 5 layers, the horizontal and vertical differences in the community structures of these low-permeability reservoirs were analyzed, and the effects of physicochemical factors such as temperature, pH, and salinity on the reservoir microbial communities on a local scale were explored. The influence of environmental change on the reservoir microbial communities at a geographical scale was discussed.

## 2. Materials and Methods

### 2.1. Sampling Site

The Block Hao41 and Block An156 samples were obtained from the Chang 8 layer of the Hujianshan Oilfield in the central and western areas of the Ordos Basin, China. At the time of sampling, the oilfield was in a water flooding period, and the injection water was comprised of processed surface water. The Block Hao41 sample was collected from the production well Hao44-5, and the Block An156 sample was collected from the production well Hu 263-47. To reduce the occasionality of the experimentation, three parallel samples were set in each group and designated as H441, H442, and H443 and A261, A262, and A263, respectively. The water cut of the samples was extremely low, and so the samples usually could be considered as pure oil samples. Even if they contained some water, it would be separated during the mixing process of the sample and the isooctane. Comprehensive reservoir information is presented in Table 1. The data for Block 154 are cited from Bian et al., 2023 [26]. They studied the differences between water and oil samples from the same well, and they separated the oil and water using a gravity difference method. Only the microbial communities of the petroleum samples were used for the comparison with the samples of this study to illustrate the differences in the vertical microbial communities.

### 2.2. Sample Collection and Pretreatment

**Figure 1:**
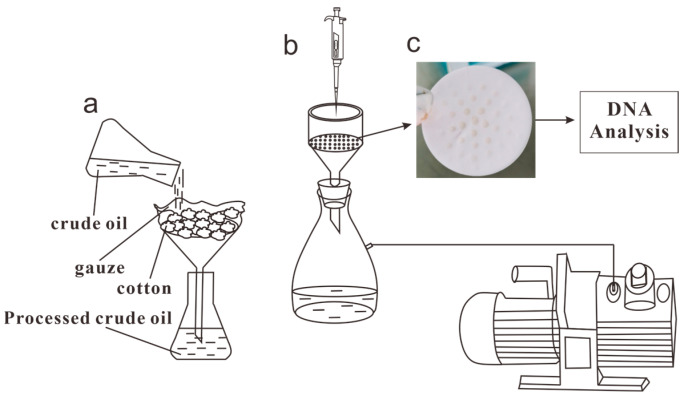
Experimental procedure: (**a**) the filtration device, (**b**) the vacuum filtration device, and (**c**) a filter with microorganisms.

The samples were obtained on 19 September 2022, and the oil samples were collected from the wellheads of Well 44-5 and Well 263-47, which were located 20 km apart. The samples were collected using sterile buckets that were sealed to prevent contamination. The experimental treatment method used for the samples was established by Wang et al. [27]. The experimental procedure is shown in Figure 1, where 200 mL of each sample were mixed and shaken with twice the volume of isooctane (2, 2, 4-trimethylpentane) and left overnight at room temperature (approximately 20 °C). The mixtures were then centrifuged at 5000× *g* for 60 min at 4 °C. After collecting the supernatants, the precipitates were dissolved again with isooctane, fully shaken, and centrifuged again under the same conditions for 30 min to remove the precipitates. The supernatants were collected twice. Sterile water was then used to moisten the filters (Delvstlab, Haining, China) and the vacuum extractor nozzle (Feiyue, Shanghai, China). Due to impurities in the crude oil samples, 0.45 µm filters were less likely to clog, resulting in samples with higher community richness than those that used 0.2 µm filters. Therefore, 0.45 µm filters were used for the centrifuged supernatants. This process separated the bacterial communities, which remained on the surfaces of the filters. The filters were then rolled up and placed in centrifuge tubes, which were stored at −20 °C.

All operations were repeated three times. The entire experiment was placed in the clean bench (SUNNE, Shanghai, China) and subjected to multiple alcohol disinfections. All equipment used was sterilized to ensure that there was no contamination from exogenous bacteria.

### 2.3. DNA Extraction and PCR Amplification

In this study, DNA extraction of the samples was performed using an E.Z.N.A.^®^ Soil DNA kit (Omega Bio-tek, Norcross, GA, USA). The extracted DNA were then examined for quality and purity using 1% agarose gel electrophoresis. The primers used for amplifying the V1-V9 region of the bacterial 16S rRNA gene were 27F (5′-AGRGTTYGATYMTGGCTCAG-3′) and 1492R (5′-RGYTACCTTGTTACGACTT-3′) [28,29]. This method provides more accurate and complete baseline data by capturing a comprehensive picture of the species’ germline information. A barcode, consisting of an eight-base sequence unique to each sample, was incorporated. The PCR reaction was performed in 4 μL of a 5 × 20 μL mix that included FastPfu Buffer, 2 μL of 2.5 mM dNTPs, 0.8 μL (5 μM) of each primer, 0.4 μL of FastPfu polymerase, and 10 ng of template DNA. The PCR procedure included initial pre-denaturation at 95 °C for 3 min, followed by 27 cycles at 95 °C for 30 s, 55 °C for 30 s, 72 °C for 30 s, and an extension at 72 °C for 10 min. The purified PCR products were prepared for Pacbio sequencing by repeating the PCR and performing polymerization and purification using an AxyPrep DNA Gel Extraction Kit (AxyPrep Biosciences, Union City, CA, USA).

### 2.4. Library Construction and Sequencing

The SMRTbell library was prepared from amplified DNA using the blunt ligation method, which was provided by the manufacturer of Pacific Biosciences. The SMRTbell libraries purified from the Zymo and HMP mock communities were sequenced on dedicated PacBio Sequel II 8M cells using a Sequencing Kit 2.0 for the chemistry. Sequencing on individual PacBio Sequel II cells allows for the purification of the SMRTbell libraries from pooled and barcoded samples. All amplicon sequencing services were provided by Shanghai Biozeron Biotechnology Co., Ltd. (Shanghai, China).

### 2.5. Processing of Sequencing Data

To obtain Cyclic Consensus Sequencing (CCS) for the demultiplexing, the PacBio platform based on SMRT sequencing technology was used. The raw reads were processed using SMRT Link Analysis Software version 9.0, which optimized the generation of the CCS reads by setting parameters such as a minimum pass count and a minimum prediction accuracy. The raw reads were processed through the SMRT portal to remove sequences that were less than 800 bp or greater than 2500 bp in length. For sequences containing 10 consecutive identical bases, barcodes, primer sequences, and chiral sequences were removed and further filtered.

Based on the clustering criterion of 98.65% similarity for the operational taxonomic units (OTU), various diversity indices analyses and statistical analyses of the community structures could be performed at each taxonomic level. The Shannon diversity index, Simpson diversity index, Chao1 diversity index, and ACE diversity index were used to analyze the species diversity within the communities. This method not only facilitated the acquisition of high-quality sequences but also enabled a comprehensive and in-depth study of the community structures.

## 3. Results

### 3.1. Sequencing Data Analysis

**Figure 2:**
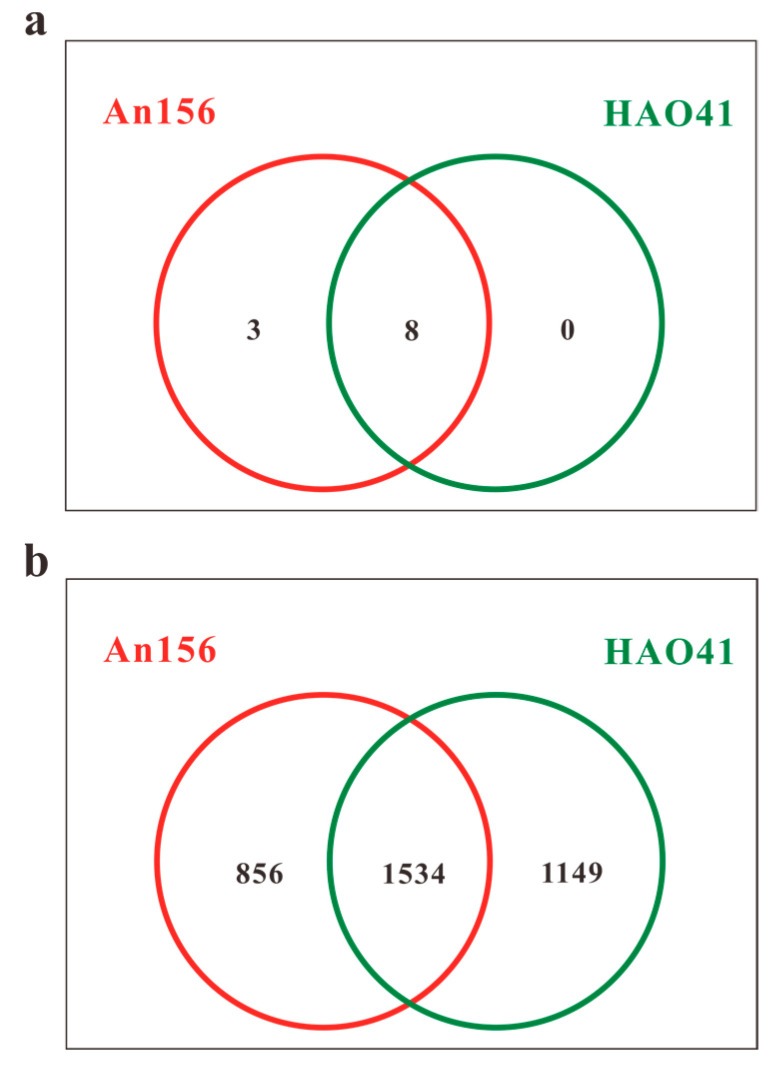
OTU Venn diagram at the phylum (**a**) and genus (**b**) levels (the numbers shown are the averages of three parallel samples).

Based on the advantages of long read lengths and a high success rate, the most mature third-generation sequencing platform was adopted in this study [30]. The original PacBio reads were processed using SMRT Link Analysis software version 9.0 to obtain the CCS. A total of 15,879 valid sequences were obtained from the samples in Block An156, with a total of 22,745,556 bp (base pairs) and an average length of 1432 bp. From the samples in Block Hao41, 18,605 effective sequences were obtained, with a total of 26,651,836 bases and an average length of 1432 bp. The UCHIME software (version 7.1) was used to cluster the OTUs with 98.65% similarity, and chimeric sequences were identified and removed using UCHIME. A total of 3539 OTUs were generated from the two sets of samples in the Chang 8 layer (Figure 2b), with 1534 OTUs shared between the two blocks. Block Hao41 had 293 more OTUs compared to Block An156. The taxonomic analysis of the representative sequences of the OTUs was performed using the uclust algorithm (version v1.2.22q), and the community composition of each sample was determined at each taxonomic level. A total of 10 phyla, 21 classes, 44 orders, 74 families, 139 genera, and 346 species were discovered.

### 3.2. Richness and Diversity of the Communities

The richness and diversity of the microbial communities could be reflected by an alpha diversity analysis. The bacterial diversity index was obtained according to the number of bacterial OTUs. The Chao and Ace indexes were used to indicate the richness of the community species. The Shannon index and Simpson index represented the diversity of the bacterial communities. They not only reflected the species richness but also indicated the species evenness [31,32,33]. As shown in Table 2, in general, the ACE index and Chao index of the bacteria in Block Hao41 were higher than those in Block An156, indicating a higher community abundance in Block Hao41. The average Shannon index of Block An156 was 6.947, which was lower than that of Block Hao41 (7.355). The Simpson index for Block An156 was 0.033, which was slightly higher than that of Block Hao41 (0.031), indicating that the community diversity in Block An156 was lower than that in Block Hao41. The data for Block 154 (A4O1 and A4O2) were cited from the study by Bian et al. in 2023 [26].

### 3.3. Analysis of the Differences between the Groups

**Figure 3:**
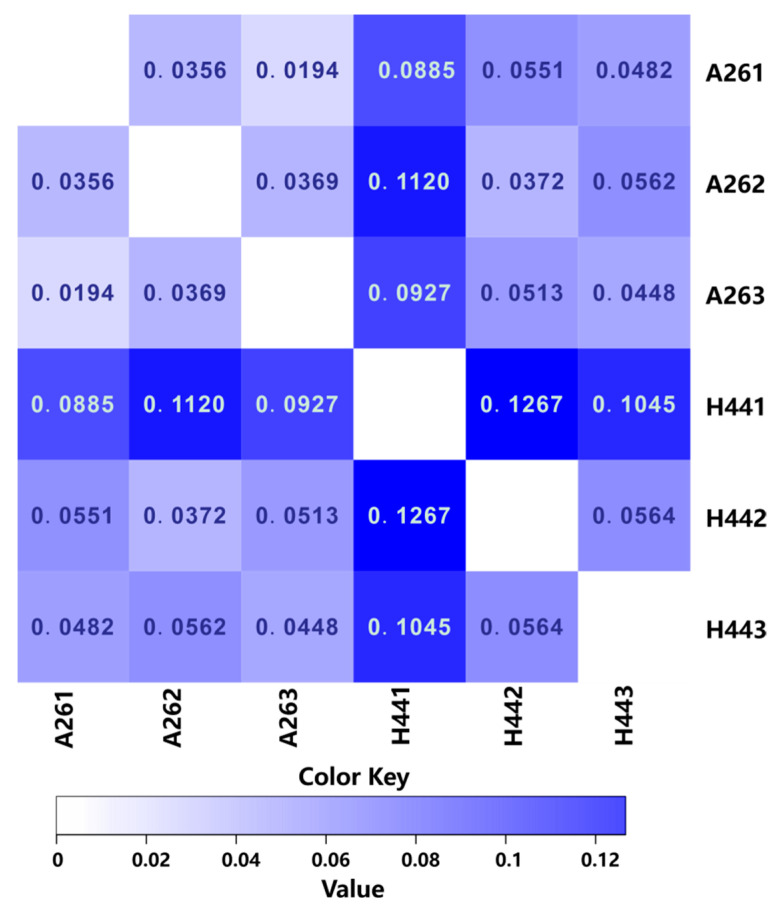
Heatmap of the distance between the samples (dissimilarity of the communities).

By using statistical algorithms to calculate the difference between two samples, a distance matrix was obtained. This matrix could be used for a beta diversity analysis and a visual statistical analysis to assess the differences in the species or functional abundances between the samples. The distance matrix was represented by a heatmap (Figure 3), which allowed for a visual examination of the differences between the samples. The color of each cell in the heatmap represents the magnitude of the data values at the intersection of the corresponding row and column variables. The Bray–Curtis dissimilarity was bound between zero and one, where zero indicated that the two groups had the same composition (that is, they shared all species) and one indicated that the two sites did not share any species. The bluer the color, the closer the number was to one, indicating that the farther the distance between the samples, the lower the similarity. From the heatmap, it could be observed that H441 exhibited significantly larger errors compared to H442 and H443, suggesting that the discrepancies in the H441 results may have been due to operational errors. Overall, the results were primarily dominated by H442 and H443. The small distance and minimal differences observed between the parallel samples (A261, A262, and A263 and H442 and H443) indicated reliable experimental results.

### 3.4. Analysis of the Microbial Community Composition

#### 3.4.1. Microbial Composition at the Phylum Level

**Figure 4:**
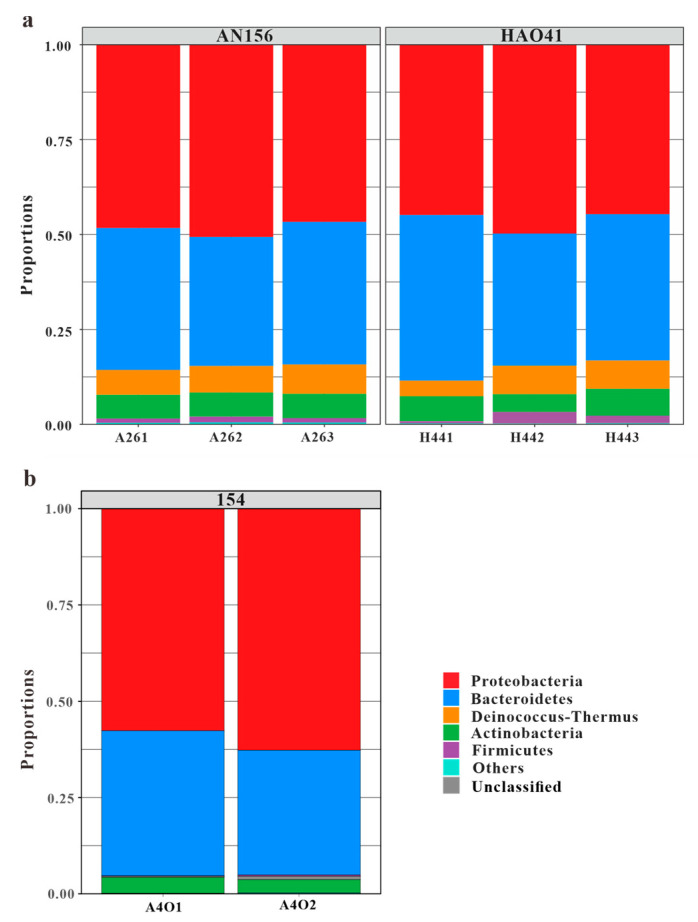
Dominant phyla in blocks An and Hao (**a**) and Block 154 (**b**) (cited from Bian et al., 2023 [26]).

A total of 10 bacterial phyla were detected in the two sample groups. Among these, five phyla with higher abundances were found to be identical in both blocks, and their proportions were similar (Figure 4a). The community was dominated by members of the following five phyla: *Proteobacteria*, which accounted for 48.5% and 46.4%, respectively; *Bacteroidetes* (36.3% and 39.0%, respectively); *Deinococcus-Thermus* (7.1% and 6.3%, respectively); *Actinobacteria* (6.4% and 6.1%, respectively); and *Firmicutes* (1.3% and 1.8%, respectively). According to the Venn diagram (Figure 2a), all phyla were present in Block An and eight phyla were shared in Block An and Block Hao. *Acidobacteria*, *Planctomycetes*, and *Verrucomicrobia* were the only three phyla that were exclusive to Block An156. However, they made up less than 1% of all bacteria in this block.

#### 3.4.2. Microbial Composition at the Genus Level

**Figure 5:**
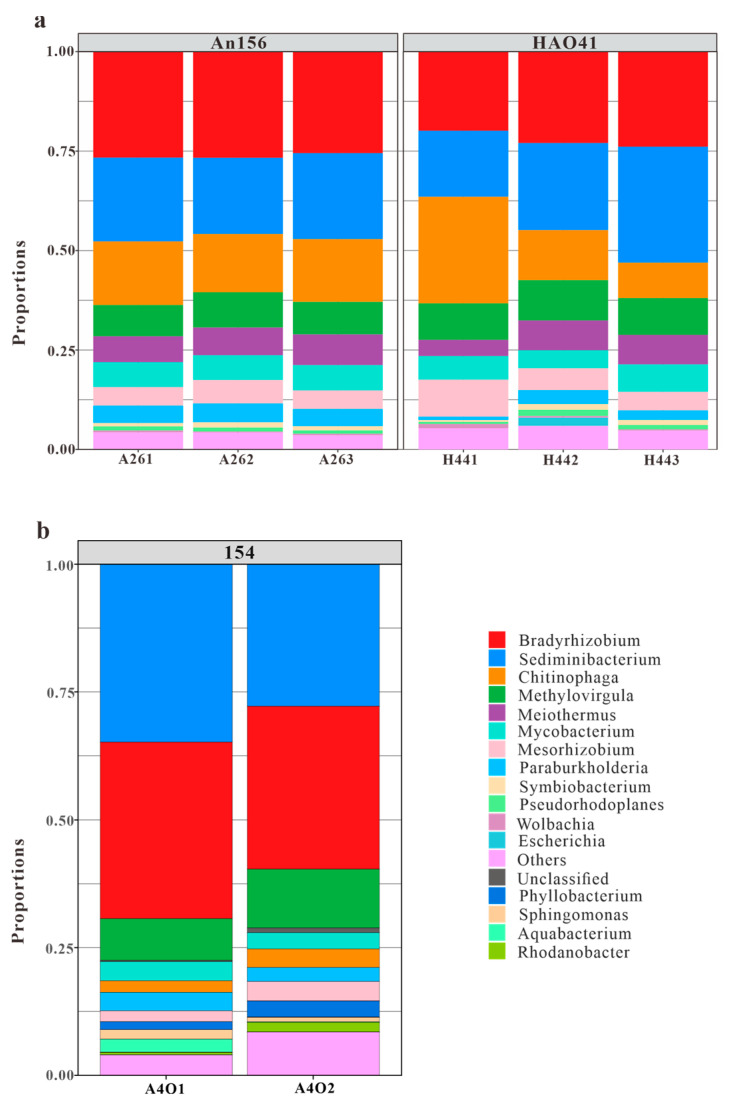
Dominant genera in blocks An and Hao (**a**) and Block 154 (**b**) (cited from Bian et al., 2023 [26]).

A total of 139 genera were detected in the samples from Block An156 and Block Hao41 (Figure 5a). In H441, *Chitinophaga* accounted for 26.8%, which was significantly higher than H442 (12.6%) and H443 (8.9%). However, the differences between H442 and H443 were small. This, again, confirmed that there were errors in H441 and that H442 and H443 were more accurate. The dominant genera were *Bradyrhizobium* (26.3% and 23.4%, respectively), *Sediminibacterium* (20.6% and 22.6%, respectively), *Chitinophaga* (15.4% and 16.1%, respectively), *Methylovirgula* (8.3% and 9.7%, respectively), *Meiothermus* (7.1% and 7.5%, respectively), *Mycobacterium* (6.3% and 5.7%, respectively), *Mesorhizobium* (5.9% and 5.1%, respectively), *Paraburkholderia* (4.5% and 2.9%, respectively), *Symbiobacterium* (1.1% and 1.1%, respectively), and *Pseudorhodoplanes* (1.0% and 1.1%, respectively).

## 4. Discussion

### 4.1. Differences in the Microbial Community Structures in the Horizontal Direction

The Ordos Basin is a part of the North China Craton, and it has a simple structure, a small structural relief, and few faults. The Hujianshan Oilfield is located in the central and western parts of the Shaanbei Slope, a secondary structural unit in the Ordos Basin. The overall structural pattern is characterized by a large and gentle westward dipping monocline [34]. Approximately 20 km separated the sampling wells horizontally in this investigation, with little tectonic changes. There was limited variation in the microbial community structures between the two wells, and comparable phyla were predominant. The phylum *Proteobacteria* (48.5% and 46.4%, respectively), phylum *Bacteroidetes* (36.3% and 39.0%, respectively), phylum *Deinococcus-Thermus* (7.1% and 6.3%, respectively), phylum *Firmicutes* (6.4% and 6.1%, respectively)*,* and phylum *Actinobacteria* (1.3% and 1.8%, respectively) were dominant. These phyla have been widely found in various petroleum reservoirs [35,36,37]. Although the unique phyla *Acidobacteria*, *Planctomycetes*, and *Verrucomicrobia* were present in Block An156, their total content was only 0.25%, which was a very small percentage. The dominant genera, including *Bradyrhizobium* (26.3% and 23.4%, respectively), *Sediminibacterium* (20.6% and 22.6%, respectively), *Chitinophaga* (15.4% and 16.1%, respectively), *Methylovirgula* (8.3% and 9.7%, respectively), *Meiothermus* (7.1% and 7.5%, respectively), *Mycobacterium* (6.3% and 5.7%, respectively), and *Mesorhizobium* (5.9% and 5.1%, respectively), showed relatively small differences in both composition and abundance. Thus, it could be considered that there was almost no difference in the community structures between the two blocks. Xu et al. [38] pointed out that the smaller the difference in pH, the smaller the difference in the bacterial communities. The results in this study showed that the pH difference in the experimental well was very small, and the communities showed little difference in the horizontal direction. Their results were consistent with the findings of this study. However, at a larger spatial scale, Gao et al. [39] observed significant variations in well community structures across different regions and formations in 22 reservoirs in China. Despite these variations, the dominant communities exhibited similarities, with *Proteobacteria*, *Bacteroidetes*, and *Actinobacteria* accounting for high proportions in most oilfields. These dominant phyla also constituted a relatively large proportion in this research block, indicating similarities in the dominant microbial communities among the oil wells. Therefore, in cases with minor structural changes, the community structure of a single well can, to some extent, represent the community structure of the entire study area.

In addition, it was found that the top 8 genera accounted for more than 80% of the total, but a total of 139 genera were identified (Figure 5a). This meant that the vast majority of genera made up only a very small fraction of the overall community. We conducted extensive literature research, and similar results were found in Gao et al. (2016) [39], Vinh (2009) [40], and Gao et al. (2019) [41]. This indicated that in an extreme environment such as an oil reservoir, microorganisms have unique characteristics, and only some microorganisms can better adapt to this reservoir environment while the survival of most bacterial species is inhibited.

### 4.2. Differences in the Microbial Community Structures in the Vertical Direction

The compositions of the microbial communities varied significantly in the vertical direction. Compared with the data of Block 154 published by Bian et al. [26], which were located at different layers in the same oilfield and approximately 300 m less deep, the bacterial abundances in Block An156 and Block Hao41 were significantly higher than that in Block 154, but the community diversity was relatively low (Table 2).

The phylum *Deinococcus-Thermus* showed significant differences in abundances between Block An156, Block Hao41, and Block 154, with proportions of 7.1%, 6.3%, and 0.2%, respectively (Figure 4b). Similarly, the phylum *Firmicutes* exhibited noticeable differences in abundance, with proportions of 1.3%, 1.8%, and 0.2%, respectively. The genera *Bradyrhizobium*, *Sedimentibacterium*, and *Thermovirgula* accounted for large proportions of the microbial communities from Block 154. The content of the genus *Chitinophaga,* within the phylum *Bacteroidetes,* varied significantly in the microbial communities from Block An156, Block Hao41, and Block 154, accounting for 15.4%, 16.1%, and 3.0%, respectively (Figure 5b). *Chitinophaga* was widely distributed in the environment, and members of this genus have been isolated from pine trees, soil, rhizosphere soil, roots, earthworm feces, and weathered rocks [42]. The proportion of *Deinococcus-Thermus* in the samples from Block An156 and Block Hao41 (7.1% and 6.3%, respectively) was also significantly higher than that in Block 154 (0.2%). In Block An156 and Block Hao41, one genus and four species of *Deinococcus-Thermus* were detected, primarily, *Meiothermus granaticius* (6.9% and 6.3%, respectively), which is a thermophilic microorganism with an optimum growth temperature of 45–50 °C and an optimum pH of 7.0–8.0 [43]. Both *Chitinophaga* and *Meiothermus granaticius* are nitrate-reducing bacteria (NRB). Nitrate-reducing bacteria can occasionally accumulate nitrites in a medium, and this inhibits the growth of sulfate-reducing bacteria in reservoirs into which nitrates are injected [21]. A phylogenetic analysis revealed that aerobic denitrifiers mainly belong to α-, β-, and γ-*Proteobacteria* [44].

*Symbiobacterium thermophilum*, the main genus of *Firmicutes* detected in the studied samples, was effectively characterized for the first time by Ohno et al. [45,46]. It is a microaerophilic heterotrophic bacterium that can grow under both microaerobic and hypoxic conditions, but it thrives under low oxygen tension [47]. Under anoxic conditions, it converts nitrate to nitrite. The bacterium demonstrated growth within the temperature range of 45 °C to 70 °C, and it has an optimum temperature of 60 °C and can grow at a pH of 6.0–9.0 at 60 °C, with an optimum pH of 7.5.

In oil reservoirs, temperature is the primary factor that restricts microbial growth. As observed in Table 1, the sampling sites in Block An156 and Block Hao41 were more than 300 m deeper than those in Block 154. The temperature increased with depth at an average rate of 3 °C/100 m (although significant regional geothermal gradient differences may have existed) [48]. It was speculated that the higher temperatures (9 °C) in Block An156 and Block Hao41 compared to that of Block 154 led to a decrease in the community diversity as the depth increased. The increasingly challenging conditions for microbial survival, resulting from rising temperatures and oxygen depletion, led to the higher abundances of thermophilic and strictly anaerobic strains within the microbial communities.

### 4.3. Effect of Physicochemical Properties and Other Factors on Community Composition

The differences in salinity and pH also affected the community compositions. The pH and salinity levels of Block An156 (pH of 7 and salinity of 52.4 g/L) and Block Hao41 (pH of 7 and salinity of 52.4 g/L) were higher than those of Block 154 (pH of 6 and salinity of 37.48 g/L). Lauber et al. [49] and Zeng et al. [50] found that there was a positive correlation between the abundance of *Actinobacteria* and *Bacteroidetes* and pH. Additionally, *Bacteroidetes* have been found to thrive in alkaline environments, which was consistent with our findings. In this study, it was found that there were higher abundances of *Actinobacteria* and *Bacteroidetes* in Block An156 and Block Hao41 compared to those in Block 154. *Actinobacteria* accounted for 6.4% and 6.1% in Block An156 and Block Hao41, respectively, while it accounted for 3.7% in Block 154.

Salinity also plays a crucial role in the development of microbial communities within reservoirs [51,52]. It is a significant factor that contributes to microbial diversity and community structure [53], and as environmental salt concentration increases, microbial extracellular osmotic pressure can also rise [54,55]. The alpha diversity levels of the communities in the samples from Block An156 and Block Hao41 were lower than those of Block 154. According to Zhang et al. [23], many microorganisms that are unable to adapt to osmotic stress may either die or become inactive, resulting in a reduction in microbial alpha diversity. The results of this study aligned with their findings [23].

## 5. Conclusions

In this study, the effects of horizontal and vertical distance on microbial community compositions were investigated by sampling at the same depths and comparing blocks at different depths. The results showed that dominant microbial communities in samples taken at the same depths but 20 km apart were similar. The dominant phyla were *Proteobacteria*, *Bacteroidetes*, *Deinococcus-Thermus*, *Actinobacteria*, and *Firmicutes*. The dominant genera included *Bradyrhizobium*, *Sediminibacterium*, *Chitinophaga*, *Methylovirgula*, *Meiothermus*, *Mycobacterium*, and *Mesorhizobium*. These findings suggested that there are no significant differences in community structures within a small range of structural variations. Therefore, a single well may serve as a representative of the local microbial community composition, eliminating the need for repeated sampling within a small horizontal distance (<20 km) in subsequent studies of microbial communities in tectonically stable areas. However, significant differences were observed in bacterial communities at different depths, with considerable variations in the dominant phyla. Notably, there were differences in the abundances of thermophilic genus *Meiothermus granaticius* and the microaerobic heterotrophic genus *Symbiobacterium thermophilum*. These differences were primarily influenced by reservoir temperature, pH, and salinity. Increasing depths led to higher formation temperatures, promoting the development of thermophilic and anaerobic species. The phyla *Actinobacteria* and *Bacteroides* showed preferences for alkaline pH environments, while salinity reduced the microbial alpha diversity. This study contributes a new perspective to comparative community research by providing a detailed analysis of differences in community structures within low-permeability reservoirs, both horizontally and vertically. For future work, this study will facilitate more targeted and comprehensive applications of MEOR, enrich the analysis of local geographic-scale communities, optimize MEOR techniques, and formulate reasonable measures for fully exploiting the potential of in situ microbial petroleum degradation. These efforts can enhance the success rate of MEOR and contribute to increased production rates in oilfields.

## Figures and Tables

**Table 1:** Reservoir parameters of the Block Hao41, Block An156, and Block 154 samples from the Hujianshan oilfield.

	Block Hao41	Block An156	Block 154
Lithology	sandstone	sandstone	sandstone
Porosity, %	9.14	11.63	12.54
Permeability, mD	0.63	1.19	3.6
Depth, m	2425	2412	2140
Salinity, g/L	52.4	52.4	37.48
Groundwater type	CaCl_2_	CaCl_2_	CaCl_2_
Well no.	44-5	263-47	Anjia 155–501
pH level	7	7	6

**Table 2:** Diversity index.

Sample	ACE	Chao	Shannon	Simpson	Evenness
A261	3937.453	3230.985	6.987	0.033	0.655
A262	3799.789	3392.482	6.749	0.035	0.634
A263	3539.175	3049.045	7.106	0.031	0.687
H441	3341.306	2892.249	8.479	0.018	0.779
H442	3960.094	3427.266	7.058	0.032	0.649
H443	4251.845	3553.8046	6.529	0.045	0.595
A4O1	2299.334	2213.439	7.521	0.026	0.678
A4O2	2696.015	2631.042	9.618	0.009	0.847

## Data Availability

Data are contained within the article.
